# Behavioral Responses of Western Flower Thrips (*Frankliniella occidentalis*) to Visual and Olfactory Cues at Short Distances

**DOI:** 10.3390/insects11030177

**Published:** 2020-03-11

**Authors:** Xiaoyun Ren, Shengyong Wu, Zhenlong Xing, Ruirui Xu, Wanzhi Cai, Zhongren Lei

**Affiliations:** 1 State Key Laboratory for Biology of Plant Diseases and Insect Pests, Institute of Plant Protection, Chinese Academy of Agricultural Sciences, Beijing 100193, China; renxiaoyunyouxiang@163.com (X.R.); sywu@ippcaas.cn (S.W.); 18234401284@163.com (R.X.); 2 Department of Entomology, College of Plant Protection, China Agricultural University, Beijing 100193, China; caiwz@cau.edu.cn; 3 School of Life Sciences, Henan University, Kaifeng 475004, China; longtaitou100@126.com

**Keywords:** *Frankliniella occidentalis*, visual cue, olfactory cue, short distance, trapping efficacy

## Abstract

Western flower thrips (WFT), *Frankliniella occidentalis* (Pergande), is a highly invasive pest, infesting many species of plants worldwide, but few studies have investigated the visual and olfactory cues associated with their foraging behaviors. In this study, the distance traveled by WFT to locate yellow cards using only visual cues and visual cues plus olfactory cues was studied first. Subsequently, preferences for colors (white, red, green, purple, yellow and blue) and patterns (triangle, rectangle, circle and flower-shape) over short distances were assessed with free-choice tests. Finally, as yellow was the most efficient color to catch WFT under laboratory conditions, the yellow flower-shape was used as the visual cue, and preferences between visual and olfactory cues were evaluated with dual choice tests. The results showed that the capture rate of WFT by visual cues decreased as selection distance increased, however capture rate remained higher with the addition of olfactory cues. The flower shape attracted the greatest number of WFT among all shapes tested. The combination of visual cues and extracted volatiles from flowering *Medicago sativa* L. attracted higher numbers of WFT than to the olfactory cues alone, however these were similar to visual cues alone. The presence of olfactory cues resulted in higher residence times by WFT than did the absence of olfactory cues. These results show the relative effects of visual and olfactory cues on the orientation of WFT to hosts and highlight that visual cues dominate selection behavior at short distances. These findings can be used in the development of efficient trapping products and management strategies for thrips.

## 1. Introduction

Western flower thrips (WFT), *Frankliniella occidentalis* (Pergande; Thysanoptera: Thripidae), is a highly polyphagous herbivore that damages many species of plants through feeding, oviposition and transmission of plant viruses, including tomato spotted wilt virus (TSWV) and impatiens necrotic spot virus (INSV) [1,2,3,4]. Both nymph and adult WFT can damage flowers, fruits and leaves [2]. Due to the inappropriate application of chemical pesticides (e.g., overuse and frequent use of pesticides), WFT has developed resistance to multiple pesticide types [5,6]. Additionally, the cryptic behavior, such as occupying narrow crevices within plants, makes it difficult to monitor WFT during early infestations [7]. As both visual and olfactory stimuli from hosts can impact foraging behaviors [8,9,10], researchers have carried out many behavioral studies aimed at developing highly efficient lures to monitor and control WFT, including colored sticky cards and chemical attractants [7,11,12].

The visual cues used by insects for orientation include color, shape, size and other physical cues associated with the hosts [7,13,14]. Low ultraviolet-reflective white, blue-violet, blue and yellow colors have been shown to be attractive to WFT [15,16,17]. Yellow and blue colors are extensively used to monitor and control thrips in crop production, including strawberries, roses and vegetables [12,15]. Yellow has broad-spectrum attraction; it is often used in monitoring and controlling insect pests, including whiteflies and aphids [18,19,20] and is attractive to thrips as well [21,22,23]. Blue is effective at luring both WFT and *Thrips tabaci* (L.) [12,24]. With a reflective wavelength of 450 nm, blue sticky cards lure significantly more thrips in greenhouses than yellow sticky cards [24]. White is also effective in attracting WFT [12], *Frankliniella intonsa* (Trybom) and *T. tabaci* [25]. In greenhouses containing cowpea, white elicited the greatest response from *F. intonsa* among 13 colors, including blue and yellow [25]. Green, the color of most leaves, is also preferred by thrips. Bian et al. (2016) reported that lawngreen-colored traps increased captures of *Scirtothrips dorsalis* (Hood; Thysanoptera: Thripidae) in the field [26]. Cao et al. (2018) used green as the background color to enhance the attractiveness of plant volatiles to WFT [27]. In addition to color, shapes affect the behavior of thrips [28]. WFT prefers flower shapes, especially symmetrical flower shapes, over rectangles, circles and other shapes [8]; flower model traps are also efficient at luring greenhouse whiteflies [28].

Chemical volatiles can also largely affect the foraging behaviors of insects [29,30], especially plant volatiles released from flowering plants containing a series of floral chemical compounds [29,31]. In a Y-tube olfactometer test, flowering plants had profound effects on the selection behaviors of *Apolygus lucorum* (Hemiptera: Miridae), which displayed a stronger preference for plants in the flowering stage than plants in nonflowering stages [32]. Among flower-visiting insects, floral scents containing oxygenated aromatic compounds are used to recognize of flowers during foraging [30,33], and floral chemicals strongly influence the behavioral preferences of thrips. Indeed, a series of floral chemicals emitted by plants, including *p*-anisaldehyde (PA) [7,11,34], benzaldehyde [11] and *o*-anisaldehyde [11], have been shown to be attractive to WFT. Adding the chemicals to sticky cards can enhance trapping efficacy [11,12].

Although the visual and olfactory cues that WFT behaviorally respond to have been studied, the efficient range in which such cues can be detected by WFT is not well known [12]. Moreover, it is necessary to understand what mediates WFT orientation, which will be beneficial for the development of efficient trapping products and strategies for the management of thrips. Thus, we carried out the following experiments: 1) using four distances (15 cm, 30 cm, 50 cm and 100 cm) to examine the behavioral responses of WFT to cues; 2) using six colors (white, yellow, red, purple, blue and green) to screen the most preferred color by WFT; 3) using four shapes (rectangle, triangle, circle and flower-shape) to evaluate the role of shape in recognition and 4) using visual and olfactory cues to analyze the cues WFT used in behavioral responses at short distances.

## 2. Materials and Methods

### 2.1. WFT Rearing

WFT individuals were collected from *Capsicum annuum* (L.) in the Mentougou Bikun Vegetable Planting Center, Beijing and reared on pods (18–25 cm) of *Phaseolus vulgaris* (L.) in an incubator (MLR-351H, Sanyo, Japan). The incubator was set at 26 ± 1 °C with a light:dark photoperiod of 12:12 h.

### 2.2. Distance Effect on Cue Perception

**Figure 1:**
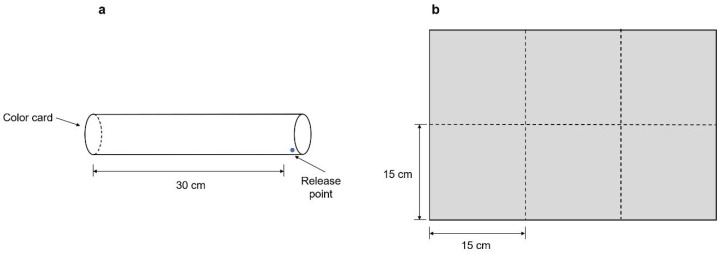
Design of the cylinder (no-choice, (**a**)) and black cardboard (free-choice, (**b**)) used for evaluation of visual cues in orientation behaviors. A cylinder of 30 cm is shown, and other experimental cylinders only differed in length. Cardboard used for testing color preferences in free-choice, and all of the sticky cards were cut into squares (15 cm × 15 cm), and were randomly pasted on the black cardboard.

The effect of distance was examined using transparent acrylic cylinders with lengths of 15 cm, 30 cm, 50 cm and 100 cm with visual cues alone and the combination of visual cues plus olfactory cues in no-choice experiments (Figure 1a). The inner and outer diameters were 6 cm and 6.5 cm respectively with a 5-cm-long adaption range. The cylinder was kept in a black box lighted with a fluorescent lamp (28 W) hung 50 cm above the cylinder. The visual cue was a 5-cm-diameter yellow sticky card (V), and 10% *p*-anisaldehyde (PA; v/v; Sigma-Aldrich, Oakville, Canada) diluted with hexane (Fisher, Fairlawn, NJ, USA), which is reported to be attractive to WFT [11], was applied. Next, 10 μL of the diluted solution was sprayed onto sticky cards (V+PA). Thirty adult WFT individuals (approximately 70% females) that were 24 h old and starved for 4 h were introduced into the cylinders from the release point (Figure 1a). Two hours later, the number of WFT captured on the sticky card was counted. Each distance experiment was replicated 10–12 times.

### 2.3. Color Preference

**Figure 2:**
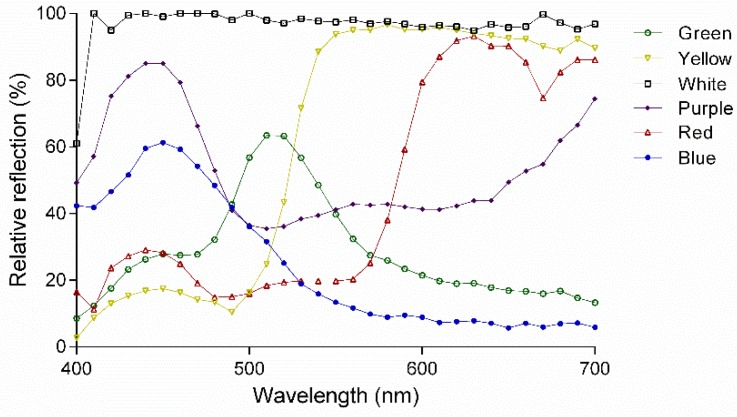
Spectral reflectance of color sticky cards used.

The red, yellow, blue, green, purple and white sticky cards used in this assay were purchased from Jiaduo Group (Hebi, China). The reflectance patterns (Figure 2) of the colored cards used in this study were measured using a spectrometer (ASD FieldSpec HandHeld, Analytical Spectral Devices Inc., Boulder, CO, USA).

The colored sticky cards were cut into square 15 cm × 15 cm shapes and randomly pasted onto black cardboard (Figure 1b). In the laboratory, the cardboard was hung on one side of a black box (60 cm × 60 cm × 60 cm), and a fluorescent lamp was hung 15 cm above the box. A total of 100 mixed-sex adults (approximately 70% females) reared on bean pods were collected for this free-choice selection [12]. After starvation for 4 h, WFT were released at a distance of 50 cm in front of the black cardboard. Two hours later, the number of WFT on each colored card was recorded; 12 replicates were conducted. Eggplants (*Solanum melongena* L.; Yuanza 5, China Vegetable Seed Technology Co. Ltd., Beijing, China) were planted for the greenhouse experiment, and WFT were identified on plants at the four-leaf stage. Plants (approximately 1.2 m) with flowers were used. Only flying WFT were assessed. The cardboard described above was hung above the eggplant canopy and placed over 4 blocks (150 m^2^/block), each containing 3 cardboard pieces. Each block served as one replicate. Two hours later, the number of thrips on the sticky cards was counted.

### 2.4. Geometric Preference Assay

As the chrysanthemum flower model trap has been shown to be effective in luring thrips [28,35] and symmetrical flowers are highly attractive to thrips [11], flower shapes with 8 petals similar to chrysanthemum flowers were used in this study. A circle shape is also preferred by thrips [7,22], and rectangles and triangles are similar in shape to the tender leaves that thrips prefer [36]. Therefore, geometric selection assays were conducted with circle, rectangle, triangle and flower shapes; the shapes were approximately 20 cm^2^ [35]. Different shapes of the same color were tested. Four different shapes of the same color were randomly hung in a square cage (50 cm × 50 cm × 50 cm), and 100 mixed-sex thrips were released from the front of the cage. Two hours later, the number of thrips on each shape was recorded. Shapes in white, yellow and blue were tested, and each color assay was replicated 10 times.

### 2.5. Visual Cues Versus Olfactory Cues at A Short Distance

The effect of visual and olfactory cues on short-distance behavioral responses was tested with a Y-tube olfactometer. The inner diameter of the Y-tube olfactometer was 1 cm with two 10-cm-long arms and a 45° connection at the 10-cm-long base tube. Air was supplied with a pump (QC-1B, Beijing Municipal Institute of Labor Protection, Beijing, China), cleaned by activated charcoal and then passed through a gas-washing bottle with distilled water. Finally, the airflow passed vials at the base tube. Medical silica gel tubes (8 mm × 9 mm; Beijing Ruiheng Junan Technology Co. Ltd., Beijing, China) were used to link each section, and the volume of airflow was 300 mL/min per arm. The visual cue (V) used was the yellow flower-shape, as yellow was the most efficient color for luring thrips based on the laboratory experiment, and the flower shape was more attractive than the other shapes. The olfactory cues (O) consisted of volatiles extracted from flowering *Medicago sativa* (L.; see Section 2.6), which is one of the preferred hosts of both adult and larval WFT [4]. Volatiles (10 μL) added to the yellow flower-shapes were used in combination of visual and olfactory cues (VO). The controls included the following: C1, the use of pure hexane; C2, the use of black cards; and C3, the use of hexane combined with black cards. Seven sets of behavioral selections were tested: (1) C1 and PA (*p*-anisaldehyde); (2) C1 and O; (3) C2 and V; (4) V and O; (5) C3 and VO; (6) V and VO and (7) O and VO. As approximately 70% adults reared in the laboratory were females and unmated females produce males by parthenogenesis [1,2], in order to better understand visual and olfactory cues in behavioral responses of female individuals, which greatly contribute to population growth, only female adults were tested [11] in this trial. Adults aged 24 h that were starved for 4 h were used, and 60 females were tested individually [7,23] for each experimental set. Observations were made on WFT preferences to cues and residence times on the selected cues for 5 min. If an adult made no choice within 3 min, it was discarded and recorded as no response.

### 2.6. Plant Volatile Collection and Analysis

*Medicago sativa* plants were grown in pots (diameter: 21 cm, height: 20 cm) in a greenhouse at 25 ± 5 °C and 60% ± 10% relative humidity. When the plants reached the flowering stage, each plant was placed in a glass jar (diameter: 25 cm, height: 40 cm) with an air inlet sealed with a glass lid (diameter: 25 cm, height: 20 cm), with an air outlet at the top. The air was purified by activated charcoal and passed over the plant. Volatiles were collected for 24 h on 50 mg of adsorbent mesh (Tenax TA 60/80 mesh, Shanghai ANPEL Scientific Instrument Company, Shanghai, China). All of the parts were connected with Teflon tubes. Air was pushed at a flow rate of 1.0 L/min using a pump. Volatiles were extracted with 300 μL hexane. The components of the volatiles were analyzed with a gas chromatograph coupled with a mass spectrometer (GCMS-QP2010SE, Shimadzu, Japan) equipped with a Rtx-5 MS capillary column (30 m × 0.25 mm i.d. × 0.25 μm, Agilent Technologies Inc. Santa Clara, CA). The injector and transfer line temperatures were 250 °C, and the ion source temperature was 230 °C. The GC oven temperature cycle was 40 °C for 1 min, a ramp to 250 °C at a rate of 8 °C/min, and a hold for 5 min. The MS scan range was 50–650 atomic mass units. Chemicals were identified by comparison to a mass spectrum library (NIST 2011, National Institute of Standards and Technology, Gaithersburg, MD, USA). The relative amounts of the chemicals identified were calculated by comparison of individual peak areas to the total peak areas of identified compounds.

### 2.7. Data Analysis

For the no-choice experiments, the numbers of captured WFT were analyzed via one-way analysis of variance (ANOVA) in conjunction with Tukey’s honestly significant difference (HSD) test for each cue at different distances to evaluate the distance effect on the perception of cues. Similarly, the number of free choices of WFT in response to colors and shapes were analyzed with one-way ANOVA, and the means were compared by Tukey’s HSD. In dual-choice tests, preferences for visual or olfactory cues were analyzed using the Chi-square test, with a 50:50 distribution. A *t*-test was applied to analyze residence times associated with preferences for cues. Data were tested for normality before analysis, and data sets of color and shape preferences were transformed by log (x+1) to meet the assumption of normal distribution and homogeneity of variance. All analyses were conducted with SAS 9.2 (SAS Institute Inc. Cary, NC, USA).

## 3. Results

### 3.1. Distance Effect on Cue Perception

**Figure 3:**
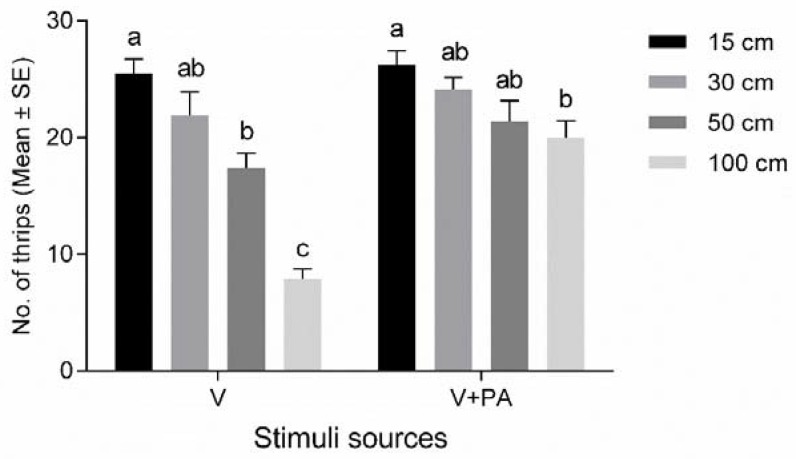
Choice of cues by western flower thrips, *Frankliniella occidentalis* at different distances. Yellow sticky card (visual cue, V) and *p*-anisaldehyde (PA) sprayed to yellow sticky card (V + PA) used in luring thrips at different distances. Different letters of the same stimulus mean orientation efficacy differed between distances (Tukey’s HSD test, *p* < 0.05).

The effect of the visual cue (yellow sticky card, V) decreased as the distance increased (*F*_3, 42_ = 37.0, *p* < 0.001; Figure 3). At short distances (≤30 cm), the visual cue was highly attractive, resulting in the capture of 25.5 ± 1.2 and 21.9 ± 2.0 WFT at 15 cm and 30 cm, respectively. As the distance increased, the capture rate of WFT decreased significantly; particularly, only 7.8 ± 0.9 individuals were captured at 100 cm. Likewise, selection frequencies for the combination of colored sticky cards and *p*-anisaldehyde (PA; V + PA) decreased with increasing distance (*F*_3, 42_ = 3.9, *p* = 0.016). However, PA appeared to increase the luring effect of the sticky cards at relatively long distances; specifically, 20.0 ± 1.4 WFT were captured at a distance of 100 cm.

### 3.2. Visual Cues Used in Short Distances

#### 3.2.1. Color Preference

**Figure 4:**
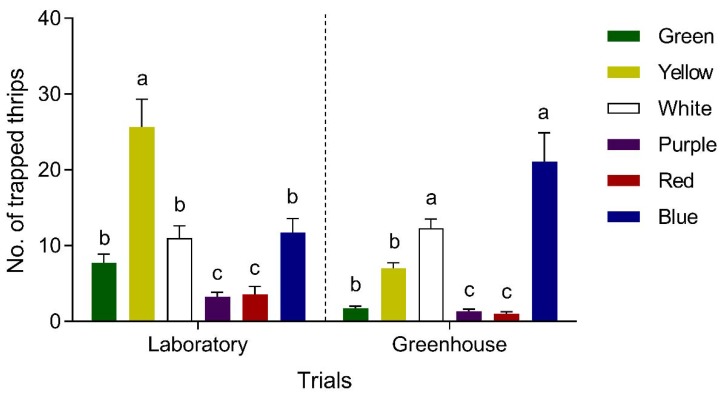
Color preferences by western flower thrips, *Frankliniella occidentalis* in the laboratory and greenhouse. Different lowercase letters indicate significantly different preferences by thrips to different colors (Tukey’s HSD test, *p* < 0.05).

In free-choice tests, WFT preferred yellow and blue to the other colors in the laboratory and greenhouse, respectively (Figure 4). Yellow sticky cards caught the most WFT (25.7 ± 3.7 WFT, accounting for 41.1%) and had the greatest luring efficacy among the six colors tested in the laboratory (*F*_5, 71_ = 17.8, *p* < 0.001), followed by blue (11.8 ± 1.8 WFT, accounting for 18.5%), white (11.0 ± 1.6 WFT, accounting for 17.5%) and green (7.7 ± 1.8 WFT, accounting for 12.3%). In the greenhouse, WFT preferred blue and white over the other colors, capturing 21.1 ± 3.8 (accounting for 43.8%) and 12.3 ± 1.2 (accounting for 27.7%) WFT, respectively (*F*_5, 23_ = 68.9, *p* < 0.001). Blue and white captured significantly more WFT than yellow (7.0 ± 0.7 WFT, accounting for 16.3%) and green (1.7 ± 0.3 WFT, accounting for 3.9%). Although no difference was observed between blue and white (*p* > 0.05), the mean number of WFT caught by blue was more than that of white. Purple and red were the least attractive to WFT both in the laboratory and greenhouse.

#### 3.2.2. Geometric Preference Assay

**Figure 5:**
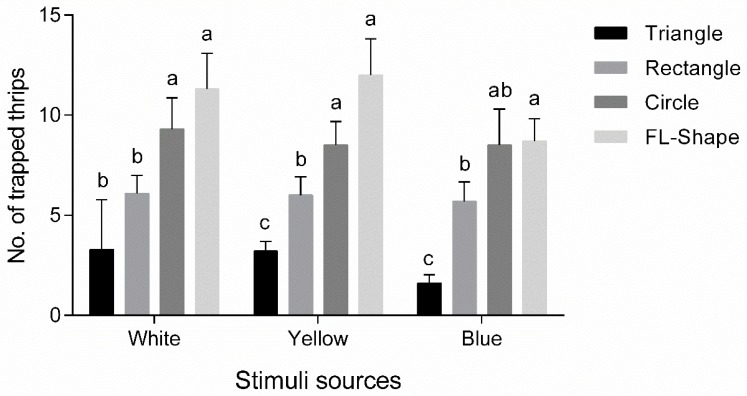
Preference of western flower thrips, *Frankliniella occidentalis* to triangle, rectangle, circle and flower shape. FL-Shape: flower shape. Different lowercase letters indicate significances between different shapes (Tukey’s HSD test, *p* < 0.05).

Preferences by WFT to geometric patterns differed and were independent of colors (white: *F*_3, 39_ = 24.7, *p* < 0.001; yellow: *F*_3, 39_ = 51.3, *p* < 0.001; blue: *F*_3, 39_ = 22.7, *p* < 0.001; Figure 5). In free-choice tests, most WFT selected the flower shape (FL-shape; white: 11.3 ± 1.8 WFT, accounting for 37.8%, yellow: 12.0 ± 1.8 WFT, accounting for 39.8%, blue: 8.7 ± 1.1 WFT, accounting for 36.4%), followed by the circular shape (white: 9.3 ± 1.6 WFT, accounting for 31.0%, yellow: 8.5 ± 1.2 WFT, accounting for 28.6%, blue: 8.5 ± 1.8 WFT, accounting for 34.7%), but no significant difference was observed between the two patterns. In addition to blue, the luring efficacy of a circle was greater than that of a rectangle (*p* < 0.05). Triangles were the least attractive to WFT. For yellow and blue sticky cards, rectangles lured significantly more WFT than triangles (*p* < 0.05), though no difference was observed between shapes when using white cards (*p* > 0.05).

### 3.3. Visual Cues Versus Olfactory Cues at A Short Distance

**Figure 6:**
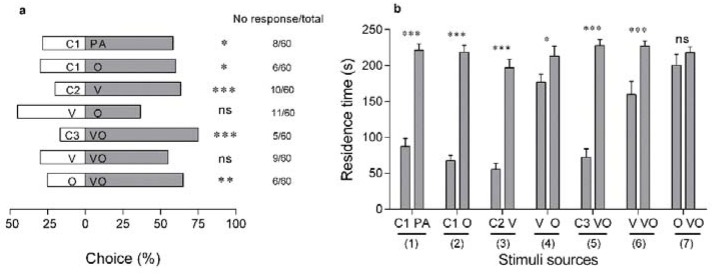
Behavioral responses of female western flower thrips, *Frankliniella occidentalis* to visual and olfactory cues. (**a**) Choice of thrips in a Y-tube olfactometer when exposed to different stimuli (Chi-square test, *p* < 0.05); (**b**) resident time of thrips in the two arms of the Y-tube olfactometer when exposed to different stimuli (*t*-test, *p* < 0.05). Visual cue (V): yellow flower- shape; olfactory cue (O): extracted volatiles from flowering *Medicago sativa*; combination of visual and olfactory cues (VO): combined volatiles of *M. sativa* with yellow flower -shape; PA: *p*-anisaldehyde; C1: hexane; C2: black card; C3: hexane combined with black card. Experiment sets: (1) C1-PA; (2) C1-O; (3) C2-V; (4) V-O; (5) C3-VO; (6) V-VO; (7) O-VO. Asterisks indicate significant differences between the stimuli (* *p* < 0.05; ** *p* < 0.01; *** *p* < 0.001); ns indicates no significant difference can be observed between treatments.

In dual-choice tests, the choice frequency (Figure 6a) and residence time (Figure 6b) of WFT with regard to visual and olfactory cues were analyzed. Both PA and plant volatiles extracted from flowering *M. sativa* had attracting effects on WFT (experiment sets 1 and 2, *p* = 0.022/0.014), and the extracted volatiles were used as the olfactory cue (O). The yellow flower-shape was used as the visual cue (V) and lured 63.3% of WFT (*p* < 0.001, experiment set 3). The residence time of WFT on O was 213.2 ± 13.9 s, significantly higher than that of WFT on V (177.1 ± 10.9 s; experiment set 4, *t*_47_ = 2.59, *p* = 0.013). Adding volatiles to yellow sticky cards (combination of visual and olfactory cues, VO) lured 75.0% of WFT, significantly more than the control (only 16.6% of WFT; *p* < 0.001; experiment set 5, Figure 6a). VO did not result in a significant increase in selection frequency compared with V alone (experiment set 6, VO: 55.0%, V: 30.0%, *p* = 0.069, Figure 6a) but did result in an increased preference compared with O alone (experiment set 7, VO: 65.0%, O: 25.0%, *p* = 0.001, Figure 6a). Nonetheless, the residence time of WFT on VO was 1.4-fold longer than that on V alone (experiment set 6, VO: 227.0 ± 7.1 s, V: 159.6 ± 18.0 s, *p* < 0.001, Figure 6b). Additionally, the residence time of WFT on VO and O was not significantly different (experiment set 7, VO: 218.2 ± 7.8 s, O: 200.3 ± 15.2 s, *p* = 0.342).

### 3.4. Plant Volatile Collection and Analysis

Twenty-two components were tentatively identified among the volatiles from flowering *M. sativa*, including floral chemicals (Table 1) such as methyl phenethyl ether, methyl benzoate, phenethyl alcohol, phenethyl acetate and methyl salicylate. In particular the most abundant component was phenethyl alcohol (29.9% ± 1.0%), followed by 1-acetoxynonane (22.0% ± 1.9%), (*Z*)-3-hexenyl acetate (17.3% ± 1.7%) and methyl phenethyl ether (10.8% ± 0.4%).

## 4. Discussion

Both visual and olfactory cues play an important role in terms of host foraging among phytophagous insects [9,13,30,37], and this can be used in the development of efficient insect management strategies [12,22,24,38]. The phototactic and chemotactic behaviors of thrips have been studied extensively, and a series of related products have been applied for crop production. However, the effective range of thrips in response to stimuli and the relative effects of visual and olfactory cues in orienting insects to hosts are largely unknown. In this study, our observations showed that at short distances (≤30 cm), visual cues dominated the behavioral responses by WFT; shape was an important factor for WFT in selection behavior, independent of color. Moreover, olfactory cues can improve orientation effects over long distances.

Distance affected visual perception by WFT in terms of location, and capture rates by colored sticky cards decreased as distance increased. At short distances (≤30 cm), WFT exhibited a strong preference for visual cues. This result was consistent with that of the host location by cabbage root flies *Delia radicum* (L.) and *Erioischia brassicae* (Bouche; both Diptera: Anthomyiidae) at close range (≤2 m); these insects can locate host plants via visual stimuli [13,39]. In another study involving in a wind tunnel (length = 1.2 m), WFT exhibited flight behavior to yellow stimuli, but the addition of odor (*p*-anisaldehyde) reduced this preference, and chemical odor was in fact shown to inhibit flight [34]. In contrast, in our study, olfactory cues combined with visual cues increased attractant efficacy at long distances (≥50 cm); as a result, significantly more WFT were attracted to the combination of *p*-anisaldehyde and yellow cards than to yellow cards alone. This finding is consistent with the hypothesis that insects use volatile chemicals (olfactory stimuli) at relatively long distances to locate hosts [12,40] but contradicts the results of the aforementioned previous study [34]. This discrepancy may be due to that study being carried out under windy conditions, which interfered with the thrips’ flying ability, resulting in the inferior efficacy of attractants [34].

When a yellow card was used, either combined with olfactory cues or used alone, it was attractive to WFT [7,34]. Visual cue is important for WFT in eliciting behavioral responses, although a prior study has indicated enhanced attraction to artificial flowers in the presence of odors [7]. Studies report numbers of thrips making choices as an index of behavioral preference [11,12,27] but few have focused on the time remaining in contact (or residence time) as an attribute of the behavioral selection [7]. Mainali and Lim (2011) found that WFT stayed longer on artificial flowers and showed active searching behaviors, indicating that WFT used the visual cues for locating hosts [7]. In the current study, when an olfactory cue was applied, whether in combination with the visual cue or utilized alone, WFT stayed longer in the presence of the olfactory cue than in its absence. Volatiles from flowering *M. sativa* were used as the olfactory cue; these volatiles contain a relatively high content of floral components [41], including methyl benzoate, phenethyl alcohol and phenethyl acetate, which have been shown to be attractive to thrips [42]. In this study, we speculated that visual cues may dominate the first choice by WFT and that olfactory cues may promote searching behavior for locating food sources by WFT.

The visual cue of shape, especially flower shape, can affect preference by WFT, and can be used for the development of more efficient colored sticky cards for monitoring and controlling thrips. The flower shape can significantly increase the WFT trapping efficacy compared with rectangular and triangular shapes, in agreement with previous studies [7,17,22,35]. It has been reported that both WFT and *F. intonsa* prefer artificial flower traps, especially symmetrical flower shapes, over other patterns [7,35]. The greenhouse whitefly *Trialeurodes vaporariorum* (Westwood) also exhibits a preference for yellow flower model traps, resulting in a capture of more than 1.8-fold than the number caught when using rectangular yellow sticky cards [28]. WFT exhibited a strong preference for flower shape, independent of color, while the greater attraction to flower shapes could be related to the higher length of edges provided by that shape compared to the others, benefiting WFT searching for cryptic habitats [2,6,7]. Moreover, circles are similar in shape to fruits or flowers, and significantly more thrips gathered on circle-shaped traps than rectangle- or triangle-shaped traps, which are similar to leaf shapes [7,36].

Blue and yellow are the colors preferred by thrips, which is consistent with previous studies [12,17,21,24,43,44]. Most researchers have found that blue is more efficient than yellow in attracting thrips [24,25,45]. In our study, blue was less attractive than yellow in the laboratory, but the opposite result was observed in the greenhouse. The different preferences for colors in the laboratory and greenhouse may be affected by light intensity and environmental conditions [22,24,46]. In the laboratory, a black background was used, providing a high contrast for yellow [22]; therefore, this background likely enhanced the luring efficacies of yellow, especially under relatively low light intensity in the laboratory. The host plants (eggplant) may also have affected the impact of color, resulting in thrips preferring blue to yellow, which is in accordance with previous studies [9,39,43,47]. In addition, the physiological status and circadian rhythm can affect insect preference for colors [48,49]. Indeed, it has been reported that the expression of photoreceptor genes for ultraviolet (UV), blue and long-wavelength-sensitivity in *Helicoverpa armigera* (Hübner) is regulated by the circadian clock and nutritional status, which can affect the localization behavior of *H. armigera* [48].

## 5. Conclusions

Our results showed that both visual and olfactory cues elicited behavioral responses by WFT, that visual cues dominated the behavioral responses at short distances and that shape also played an important role in the perception of visual cues. It was observed that olfactory cues improved the efficacy of orientation over a relatively long distance. These findings may help develop efficient traps for monitoring and controlling thrips.

## Figures and Tables

**Table 1:** Tentatively volatile components of flowering *Medicago sativa*.

Compound	Retention Time	Relative Content (%) ^1^
(*Z*)-3-hexenyl acetate	8.89	17.3 ± 1.7
(*E*)-2-hexenyl acetate	9.071	0.2 ± 0.1
3-methylcyclohex-2-en-1-ol	9.317	0.4 ± 0.1
d-limonene	9.493	0.2 ± 0.1
phenylacetaldehyde	9.753	0.3 ± 0.1
(*E*)-β-ocimene	9.786	0.4 ± 0.1
methyl phenethyl ether	10.482	10.8 ± 0.4
methyl benzoate	10.782	0.1 ± 0.0
nonanal	10.937	1.2 ± 0.0
phenethyl alcohol	11.138	29.9 ± 1.0
4-methoxystyrene	11.944	0.3 ± 0.1
1,4-dimethoxybenzene	12.105	0.5 ± 0.1
(*Z*)-3-hexenyl butanoate	12.475	0.3 ± 0.0
methyl salicylate	12.731	0.9 ± 0.1
decanal	12.894	1.1 ± 0.1
ethyl nicotinate	13.083	0.4 ± 0.0
(*Z*)-3-hexenyl isovalerate	13.41	0.3 ± 0.0
phenethyl acetate	13.801	7.3 ± 0.6
2-undecanone	14.46	1.3 ± 0.1
1-acetoxynonane	14.731	22.0 ± 1.9
germacrene D	17.833	0.9 ± 0.1
2,4-di-tert-butylphenol	18.061	4.0 ± 0.1

^1^: Proportions (%) of peak areas to total areas of identified compounds.
